# Abdominal CT: a radiologist-driven adjustment of the dose of iodinated contrast agent approaches a calculation per lean body weight

**DOI:** 10.1186/s41747-018-0074-1

**Published:** 2018-12-05

**Authors:** Moreno Zanardo, Fabio Martino Doniselli, Anastassia Esseridou, Stefania Tritella, Chiara Mattiuz, Laura Menicagli, Giovanni Di Leo, Francesco Sardanelli

**Affiliations:** 1 PhD Course in Integrative Biomedical Research, Department of Biomedical Sciences for Health, Università degli Studi di Milano, Via Mangiagalli 31, 20133 Milan, Italy; 2 Postgraduate School in Radiodiagnostics, Università degli Studi di Milano, Via Festa del Perdono 7, 20122 Milan, Italy; 3 Radiology Unit, IRCCS Policlinico San Donato, Via Morandi 30, 20097 San Donato Milanese, Italy; 4 Department of Biomedical Sciences for Health, Università degli Studi di Milano, Via Morandi 30, 20097 San Donato Milanese, Italy

**Keywords:** Abdomen, Body composition, Body weight, Contrast media, Tomography (x-ray, computed)

## Abstract

**Background:**

The contrast agent (CA) dose for abdominal computed tomography (CT) is typically based on patient total body weight (TBW), ignoring adipose tissue distribution. We report on our experience of dosing according to the lean body weight (LBW).

**Methods:**

After Ethics Committee approval, we retrospectively screened 219 consecutive patients, 18 being excluded for not matching the inclusion criteria. Thus, 201 were analysed (106 males), all undergoing a contrast-enhanced abdominal CT with iopamidol (370 mgI/mL) or iomeprol (400 mgI/mL). LBW was estimated using validated formulas. Liver contrast-enhancement (CE_L_) was measured. Data were reported as mean ± standard deviation. Pearson correlation coefficient, ANOVA, and the Levene test were used.

**Results:**

Mean age was 66 ± 13 years, TBW 72 ± 15 kg, LBW 53 ± 11 kg, and LBW/TBW ratio 74 ± 8%; body mass index was 26 ± 5 kg/m^2^, with 9 underweight patients (4%), 82 normal weight (41%), 76 overweight (38%), and 34 obese (17%). The administered CA dose was 0.46 ± 0.06 gI/kg of TBW, corresponding to 0.63 ± 0.09 gI/kg of LBW. A negative correlation was found between TBW and CA dose (*r* = -0.683, *p* < 0.001). CE_L_ (Hounsfield units) was 51 ± 18 in underweight patients, 44 ± 8 in normal weight, 42 ± 9 in overweight, and 40 ± 6 in obese, with a significant difference for both mean (*p* = 0.004) and variance (*p* < 0.001). A low but significant positive correlation was found between CE_L_ and CA dose in gI per TBW (*r* = 0.371, *p* < 0.001) or per LBW (*r* = 0.333, *p* < 0.001).

**Conclusions:**

The injected CA dose was highly variable, with obese patients receiving a lower dose than underweight patients, as a radiologist-driven ‘compensation effect’. Diagnostic abdomen CT examinations may be obtained using 0.63 gI/kg of LBW.

## Key points

- Contrast agent dose based on total body weight ignores body composition
- Underweight patients received a higher dose than obese patients
- Diagnostic abdominal CT examinations may be obtained using 0.63 gI/kg of lean body weight

## Background

Factors impacting on contrast enhancement in single-energy computed tomography (CT) include concentration and dose of the iodinated contrast agent (CA), injection rate, scanning delay time, saline-solution flushing, and cardiac output [1–3]. The CA dose, expressed in gI/kg, is one of the most important factors determining the parenchymal liver contrast enhancement (CE_L_) [4].

When considering delayed scanning phases, another important factor determining CE_L_ is the CA biodistribution into the intra- and extra-vascular spaces, which are both related to the body size [5, 6]. As a consequence, if a fixed amount of iodine is administered to patients, which is still a usual practice [7], some may receive an unnecessary high dose while others may receive a suboptimal dose.

Since 2000, several studies have demonstrated smaller variations in CE_L_ when CA is dosed on the patient total body weight (TBW) instead of administering a fixed amount [8–14]. Dosing according to TBW is reasonably effective, but it can lead to overdosing obese patients or underdosing patients with high contribution of the lean body weight (LBW) over the TBW, such as athletes. Indeed, a large proportion of TBW of obese patients consists of poorly perfused adipose tissue, where the CA poorly distributes [2, 10, 15, 16], as 99% of metabolic processes take place in the LBW [17, 18].

Various body size indexes have been proposed to determine the CA dose for abdominal multiphasic or portal-venous-phase CT, demonstrating better results for LBW rather than TBW [16, 19–27]. LBW can be determined by many different methods such as dual-energy x-ray absorptiometry, CT, ultrasound, and bioelectrical impedance analysis [28], although an equation based on the patient TBW, height, and gender, or the determination by bioelectrical impedance balance is the most suitable for calculation [18].

Previous studies using LBW for CA dose calculation mainly compared different strategies and, to our knowledge, no study has performed an optimisation process to find out what is the minimal diagnostic CA dose based on the LBW [19–27]. Moreover, authors limited their studies mainly to normal-weight or overweight populations. In our opinion, advantages of CA dose calculation based on the LBW instead of the TBW may appear mostly in underweight and obese patients.

The aim of this preliminary retrospective study was to report on our experience on multiphasic abdominal CT and to calculate the LBW-derived CA dose that was equivalent to that derived from TBW, leading to the same amount of injected iodine. In other words, our final aim was to find a feasible formula to standardise the amount of CE_L_ across patients of all sizes, whereas the amount of iodine delivered will vary by patient size and by whether LBW or TBW is used to determine the total amount of injected iodine.

## Methods

### Study design and population

This retrospective cross-sectional study was approved by the local Ethics Committee (San Raffaele Hospital, Milan, authorisation number 160/int/2016). A series of consecutive patients who underwent a contrast-enhanced multiphasic abdominal CT or portal-venous-phase CT at our institution from June to September 2016 were reviewed.

Exclusion criteria were: history of chronic liver disease (cirrhosis, local or diffuse liver fatty infiltration, or glycogen storage disease); congestive heart failure; prior cardiac valve replacement; restrictive and/or constrictive pericarditis; implanted devices (pacemakers, defibrillators, insulin pumps). Although Hamer et al. [29] defined steatotic hepatitis when liver parenchyma has an average CT value on unenhanced images lower than 40 Hounsfield units (HU), we excluded patients with CT values below 30 HU in the unenhanced scan. As a consequence, low grades of steatosis have presumably been included in our study population.

### CT protocol

All patients were studied using a 64-row CT scan (Somatom Definition, Siemens Medical Solution, Erlangen, Germany) with 120 kVp, tube load from 100 to 200 mAs depending on automatic exposure control system (CARE Dose 4D, Siemens Medical Solution, Erlangen, Germany), rotation time 0.5 s, pitch 1, B30f medium smooth for kernel reconstruction technique and abdomen window.

Patients’ TBW and height were registered in an electronic database. Moreover, a radiologist-driven dose of iopamidol (190 patients over 201) (Iopamiro 370; 370 mgI/mL; Bracco Imaging, Milan, Italy) or iomeprol (11 patients over 201) (Iomeron 400; 400 mgI/mL; Bracco Imaging, Milan, Italy) was administered. While iopamidol is the main choice in our hospital for routine abdomen and chest CT, iomeprol is used for cardiac CT. Due to practical reasons (storage lack of iopamidol, the necessity of employing an already-open CA bottle, examination acquired during a cardiac session), some patients received iomeprol. A total of eight radiologists were responsible for the examinations. The general rule established in the department for the CA dose to be administered for multiphasic abdominal CT was to use doses proportional to the TBW, multiplying the patient body weight by a constant, which varied from 1.1 to 1.3 mL/kg, with adjustments when the CA dose was considered too high. Radiologists usually adopted their own spontaneous threshold, without any agreement among them. Another heuristic rule used by some professionals was ‘patient weight in millilitres plus 10 additional millilitres of CA’.

The CA was administered intravenously through a 20-gauge needle using an automatic power injector (EmpowerCTA® Contrast Injection System, Bracco Imaging, Milan, Italy) at the rate of 3 mL/s, followed by 50 mL of saline solution at the same rate.

The scan delay was determined using an automated triggering hardware and a dedicated software (Bolus Tracking, Siemens Medical Solution, Erlangen, Germany). Specifically, low-dose monitor images were obtained in a single axial slice of the aorta after CA injection. When the descending aorta enhanced more than 100 HU, diagnostic scans of the abdomen were acquired after an additional delay of about 18 s (arterial phase), 30 s after arterial phase (portal-venous phase), and, only in specific cases, 90 s (nephrogenic phase). For the aim of this study, we considered only the portal venous phase.

### LBW estimation and image analysis

According to the international classification of body mass index (BMI) from the WHO [30], patients were considered underweight when the BMI was lower than 18.5 kg/m^2^, normal weight when between 18.5 kg/m^2^ and 25 kg/m^2^, overweight when between 25 kg/m^2^ and 30 kg/m^2^, and obese when higher than 30 kg/m^2^.

According to Awai and colleagues [31] and Nyman [32], LBW was calculated using the James formula [33] (Eq. 1) or the Boer formula [33] (Eq. 2), due to better adherence of non-obese patients to the first and of obese patients to the latter:

1 $$ \kern5em {LBW}_{James}=\left\{\begin{array}{c}1.1\times weight\ \left(\mathrm{kg}\right)-128\times {\left(\frac{weight\ \left(\mathrm{kg}\right)}{height\ \left(\mathrm{m}\right)}\right)}^2\mathrm{men}\\ {}1.07\times weight\ \left(\mathrm{kg}\right)-148\times {\left(\frac{weight\ \left(\mathrm{kg}\right)}{height\ \left(\mathrm{m}\right)}\right)}^2\mathrm{women}\end{array}\right\} $$

2 $$ {LBW}_{Boer}=\left\{\begin{array}{c}0.407\times weight\ \left(\mathrm{kg}\right)+0.267\times height-19.2\ \mathrm{men}\\ {}0.252\times weight\ \left(\mathrm{kg}\right)+0.473\times height-48.3\ \mathrm{women}\end{array}\right\} $$

All images were reviewed by a radiology resident with 2 years of experience in the field of abdominal CT. Attenuation measurements were obtained by manually placing a rounded region of interest in the anterior (III or IVb Couinaud) and in the posterior (VI Couinaud) segments at the level of the main portal vein with a diameter of between 2 and 3 cm; these two values were averaged. Two different regions in anterior and posterior liver parenchyma were chosen because of subtle territorial differences in liver enhancement, probably due to different levels of fatty infiltration and vascularity. Focal hepatic lesions, blood vessels, bile ducts, calcifications, as well as artefacts, if present, were carefully avoided.

### Statistical analysis

For each patient, we retrieved from the radiological report the amount and type of injected CA in millilitres and the dose was calculated both per TBW and LBW. Moreover, to account for the different concentration of iodine of the two used CAs, we converted the absolute injected amount from millilitres to grams of iodine (gI).

The CE_L_ was calculated as the difference between the CT value measured in the portal venous phase and that measured before CA injection. To this aim, regions of interest were copy-pasted from one phase to another.

The distribution of CE_L_ was calculated for the whole population and for the four subgroups of BMI. Bivariate correlation analysis was performed using the Pearson correlation coefficient. The comparison of the mean CA dose, as well as of the mean CE_L_ among the four subgroups of BMI was performed using the one-way analysis of variance (ANOVA); the variance of CE_L_ was compared using the Levene test of homoscedasticity.

Differences in the practice of CA dose calculation among radiologists were evaluated using the ANOVA.

Continuous data were presented as mean and standard deviation while categorical data were presented as counts and percentages. The coefficient of variation (CoV) was calculated as the standard deviation/mean ratio.

Statistical analysis was carried out using SPSS Statistics (SPSS v.24, IBM Inc., Armonk, NY, USA). A *p* value < 0.050 was regarded as statistically significant.

## Results

### Distributions

A total of 219 patients were screened, 18 of whom were excluded for having chronic liver disease (*n* = 13), implanted device (*n* = 4), or congestive heart failure (*n* = 1). Thus, 201 patients were analysed, 106 men (53%) and 95 women (47%), with a mean age of 66 ± 13 years (CoV 20%), mean TBW 72 ± 15 kg (CoV 21%), mean LBW 53 ± 11 kg (CoV 20%), and mean LBW/TBW 74 ± 8% (CoV 11%).

The mean BMI was 26 ± 5 kg/m^2^ with 9 patients (4%) classified as underweight, 82 (41%) as normal weight, 76 (38%) as overweight and 34 (17%) as obese. Demographics and other data of the study population are presented in Table 1.

**Table 1:** Demographic characteristics of 201 patients of the study population

Item	Value
Total number of patients	201
Gender	106 males (53%)
Mean age (years)	66 ± 13
Mean height (m)	1.66 ± 0.10
Mean TBW (kg)	72 ± 15
Mean LBW (kg)	53 ± 11
Mean percent LBW/TBW (%)	74 ± 8
Mean BMI (kg/m^2^):	26 ± 5
Underweight (BMI < 18.5 kg/m^2^)	9 (4%)
Normal weight (18.5 ≤ BMI < 25 kg/m^2^)	82 (41%)
Overweight (25 ≤ BMI < 30 kg/m^2^)	76 (38%)
Obese (BMI ≥ 30 kg/m^2^)	34 (17%)

*BMI* body mass index, *LBW* lean bodyweight, *TBW* total bodyweight

The mean injected amount of CA was 32 ± 5 gI (CoV 16%). Expressed in terms of gI/kg of TBW or LBW, the mean CA dose was 0.46 ± 0.06 (CoV 13%) or 0.63 ± 0.09 (CoV 14%), respectively.

The mean CT value of the liver was 53 ± 8 HU before CA injection and 96 ± 13 HU in the portal venous phase, for a mean CE_L_ of 43 ± 9 HU (CoV 21%). These and other data are reported in Table 2.

**Table 2:** Mean contrast agent dose administered in 201 patients of the study population

Item	Value^a^	CoV
Amount of injected CA (g)	32 ± 5	16%
Dose of CA per TBW (gI/kg)	0.46 ± 0.06	13%
Dose of CA per TBW (gI/kg) according to BMI^b^		
Underweight (BMI < 18.5 kg/m^2^)	0.56 ± 0.08	14%
Normal weight (18.5 ≤ BMI < 25 kg/m^2^)	0.48 ± 0.05	10%
Overweight (25 ≤ BMI < 30 kg/m^2^)	0.44 ± 0.04	9%
Obese (BMI ≥ 30 kg/m^2^)	0.41 ± 0.04	10%
Dose of CA per LBW (gI/kg)	0.63 ± 0.09	14%
Dose of CA per LBW (gI/kg) according to BMI^c^		
Underweight (BMI < 18.5 kg/m^2^)	0.68 ± 0.11	16%
Normal weight (18.5 ≤ BMI < 25 kg/m^2^)	0.62 ± 0.08	13%
Overweight (25 ≤ BMI < 30 kg/m^2^)	0.62 ± 0.08	13%
Obese (BMI ≥ 30 kg/m^2^)	0.65 ± 0.11	17%
Liver contrast enhancement (HU)^d^	43 ± 9	21%
Liver contrast enhancement (HU) according to BMI^e^		
Underweight (BMI < 18.5 kg/m^2^)	51 ± 18	35%
Normal weight (18.5 ≤ BMI < 25 kg/m^2^)	44 ± 8	18%
Overweight (25 ≤ BMI < 30 kg/m^2^)	42 ± 9	18%
Obese (BMI ≥ 30 kg/m^2^)	40 ± 6	15%

^a^Data represent mean ± standard deviation

^b^This trend showed a significant negative association (*p* < 0.001)

^c^The comparison was not statistically significant (*p* ≥ 0.065)

^d^Calculated as the difference between the computed tomography (CT) value measured in the venous phase and that measured before contrast agent injection

^e^This trend showed a significant negative association (*p* = 0.004)

*BMI* body mass index, *CA* contrast agent, *CoV* coefficient of variation, *gI* grams of iodine, *HU* Hounsfield units, *LBW* lean bodyweight, *TBW* total bodyweight

### Correlation analysis

**Fig. 1:**
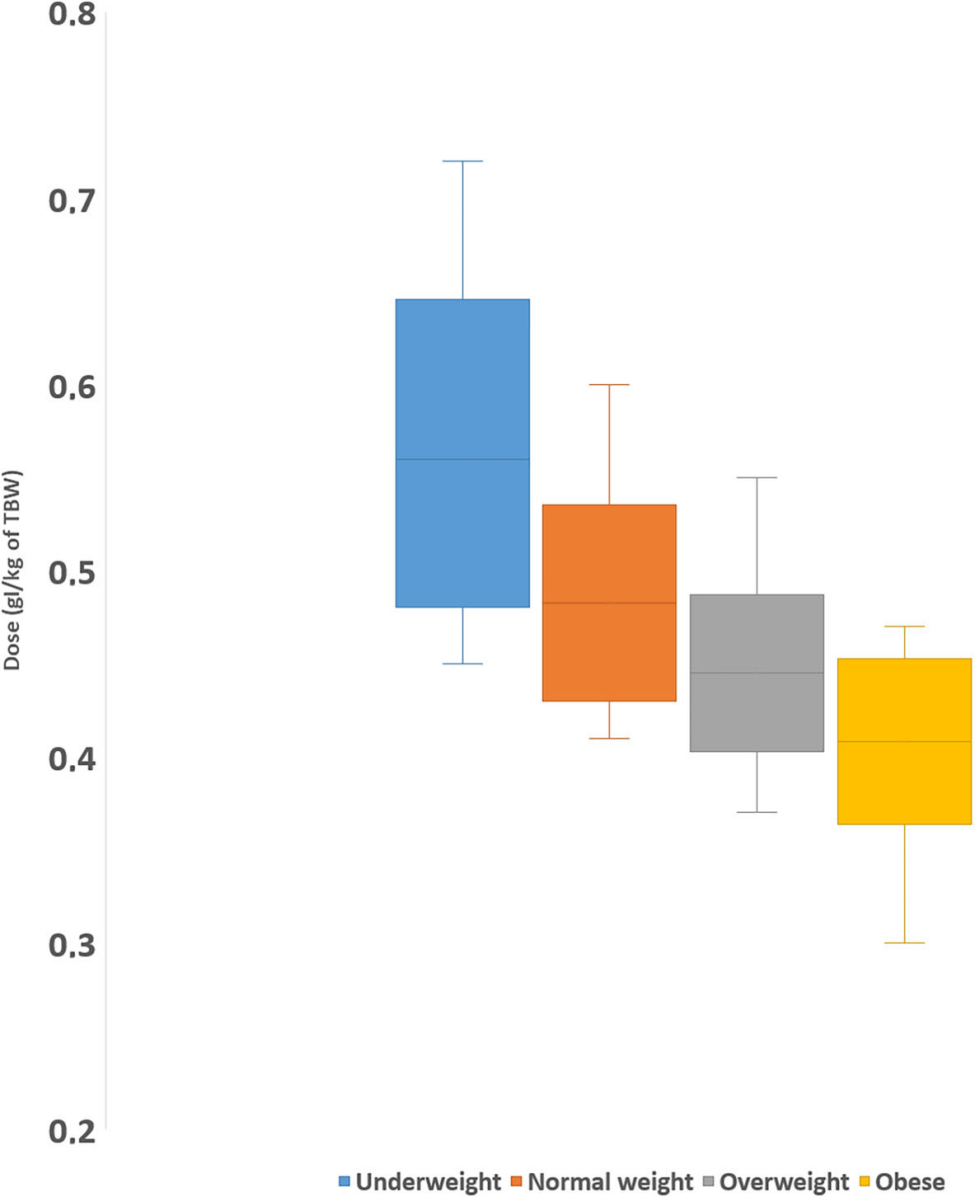
Box plot showing the mean CA dose (in grams of iodine per kilogram of total body weight) per each of the 4 subgroups of the body mass index (BMI) according the WHO (centre line). Mean ± standard deviation (box) and the minimum and the maximum values are also showed (outer lines)

A significant high negative correlation was observed between CA dose and TBW (*r* = -0.683; *p* < 0.001). In particular, the mean CA dose was 0.56 ± 0.08 gI/kg for underweight, 0.48 ± 0.05 gI/kg for normal-weight patients, 0.44 ± 0.04 gI/kg for overweight patients, and 0.41 ± 0.05 gI/kg for obese patients (*p* < 0.001) (Fig. 1).

A low but significant negative correlation was found between CE_L_ and TBW (*r* = -0.292; *p* < 0.001) or LBW (*r* = -0.316; *p* < 0.001). A low but significant positive correlation was found between CE_L_ and CA dose in gI/kg of TBW (*r* = 0.371, *p* < 0.001) or in gI/kg of LBW (*r* = 0.333, *p* < 0.001).

A low but significant negative correlation was found between CE_L_ and BMI (*r* = -0.206; *p* = 0.003). According to the four subgroups of BMI, the mean CE_L_ was 51 ± 18 HU (CoV 35%) in underweight patients, 44 ± 8 HU (CoV 18%) in normal-weight patients, 42 ± 9 HU (CoV 18%) in overweight patients, and 40 ± 6 HU (CoV 15%) in obese patients with a statistically significant difference of both the mean (*p* = 0.004) and variance (*p* < 0.001).

The mean CA dose in gI/kg of TBW did not differ significantly among the eight radiologists, varying from 0.43 to 0.47 gI/kg (*p* = 0.510).

## Discussion

In this study, we retrospectively evaluated 201 patients undergoing an abdominal CT at our institution. The main finding was the evidence of a kind of ‘compensation effect’, subjectively operated by the radiologists, on the injected CA dose, always considering its calculation in terms of gI/kg. In fact, we observed a deviation from proportionality between the TBW and the injected CA dose that they declared when questioned about how they decide CA dose. This was clear in particular for obese patients, which received a CA dose lower than that theoretically based on the TBW. Indeed, a high negative correlation was found between the patient TBW and the administered CA dose, meaning that the higher the TBW, the lower the CA dose.

Although there are no recommendations for obese patients (apart from a general limit of 250 mL reported on the drug information sheet), radiologists preferred to apply a kind of precautionary principle by subjectively reducing CA dose in obese patients. We could speculate that radiologists have somehow weighted the CA dose according to a plausible LBW, even without being aware of the exact LBW.

Importantly, although the CA dose was reduced in obese patients, all CT examinations were judged as diagnostic and no patients received a repeat examination. This allows us to hypothesise a margin for dose reduction, especially in underweight patients, who in our study received a dose in gI/kg significantly higher than obese ones. In fact, the other side of the above-mentioned compensation effect is a potential overdosing in underweight patients, partially due to the fear of a non-diagnostic examination if injected strictly according to the TBW.

Another result of this study is a trend toward a lower and lower variability of CE_L_ from underweight to obese patients (Levene test *p* < 0.001). We speculate that this evidence indirectly demonstrates that LBW could be better suited for dosing CA than TBW. In fact, poor but non-negligible CA perfusion in the adipose tissue would only increase variability of CE_L_.

Interestingly, the practice of adjusting the CA dose, unconsciously weighting for the LBW, was observed in all the staff radiologists, without significant differences among them. Of note, this shared practice spontaneously evolved in the department real-life. In addition, repeat CT examinations due to insufficient parenchymal contrast-enhancement has never been reported.

We also obtained an equivalent CA dose, *i.e.* the CA dose based on the LBW that would have provided the same amount of iodine that was actually administered. In the study population, a mean of 32 ± 5 gI was injected, corresponding to 0.46 ± 0.06 gI/kg of TBW or 0.63 ± 0.09 gI/kg of LBW. Although the latter data were back calculated from the raw data, it is reasonably to think that if 0.63 gI/kg of LBW had been used instead of 0.46 gI/kg of TBW, it would have resulted in an equivalent mean CE_L_.

The use of LBW instead of TBW on a patient-by-patient base could impact on the overall CA dose to the population, permitting a more personalised approach to CA administration and a possible reduction of the total amount of CA administered to the population. Moreover, as obesity increases the risk for kidney disease [34], a reduction of the overall dose to obese patients represents a further advantage in terms of risk of nephrotoxic effects from iodinated CA. The associated cost reduction, dependent on changes in administered volume, is another advantage potentially deriving from this approach [7]. However, although Awai et al. [31] have already identified LBW as the best indicator for determining the proper amount of CA, its use has never entered clinical practice.

When optimising CA dose, several factors should be taken into account [35]. From pharmacokinetics, it is known that drug distribution in the arterial phase mainly depends on heart function, while in the portal venous phase vasoconstriction-vasodilatation play the main role in determining CA distribution [18, 36]. We based our results on CE_L_ but a wide variety of para-physiological conditions can affect liver parenchyma in the general population (*e.g.* steatotic hepatitis, diffuse cirrhosis, previous liver diseases) that can affect CE_L_.

This study has limitations. First of all, its retrospective design and relatively small number of patients, which implies an uneven distribution of patient weights. In addition, we estimated the patient LBW using the James or Boer formulas. Indeed, LBW determined with the aid of a total body composition analyser may yield a more accurate analysis. There are several prediction formulas for LBW [33, 37–39] that may yield different results. However, for the aim of our study, the formulas we used are considered the simplest methods for retrospective calculation of the LBW, confirmed by Caruso et al. [40].

In conclusion, the CA dose injected at our institution for abdominal multiphasic CT was highly variable, with obese patients receiving a much lower dose than underweight patients, as a radiologist-driven ‘compensation effect’. Diagnostic abdominal CT may be obtained using 0.63 gI/kg of LBW and margins for dose reduction do exist.

## Abbreviations

BMI: Body mass index
CA: Contrast agent
CE_L_: Liver contrast enhancement
CoV: Coefficient of variation
CT: Computed tomography
HU: Hounsfield units
LBW: Lean body weight
TBW: Total body weight
